# Dynamics of Loneliness Among Older Adults During the COVID-19 Pandemic: Pilot Study of Ecological Momentary Assessment With Network Analysis

**DOI:** 10.3389/fdgth.2022.814179

**Published:** 2022-02-07

**Authors:** Varsha D. Badal, Ellen E. Lee, Rebecca Daly, Emma M. Parrish, Ho-Cheol Kim, Dilip V. Jeste, Colin A. Depp

**Affiliations:** ^1^Department of Psychiatry, University of California, San Diego, San Diego, CA, United States; ^2^Department of Psychiatry, Sam and Rose Stein Institute for Research on Aging, University of California, San Diego, San Diego, CA, United States; ^3^Desert-Pacific Mental Illness Research Education and Clinical Center, Veterans Affairs San Diego Healthcare System, San Diego, CA, United States; ^4^San Diego State University/University of California, San Diego Joint Doctoral Program in Clinical Psychology, San Diego, CA, United States; ^5^AI and Cognitive Software, International Business Machines (IBM) Research-Almaden, San Jose, CA, United States; ^6^Department of Neurosciences, University of California, San Diego, San Diego, CA, United States; ^7^Veterans Affairs (VA) San Diego Healthcare System, La Jolla, CA, United States

**Keywords:** aging, dynamic networks, causal networks, positive affect, negative affect, social isolation, loneliness

## Abstract

**Objective:**

The COVID-19 pandemic has had potentially severe psychological implications for older adults, including those in retirement communities, due to restricted social interactions, but the day-to-day experience of loneliness has received limited study. We sought to investigate sequential association, if any, between loneliness, activity, and affect.

**Methods:**

We used ecological momentary assessment (EMA) with dynamic network analysis to investigate the affective and behavioral concomitants of loneliness in 22 residents of an independent living sector of a continuing care retirement community (mean age 80.2; range 68–93 years).

**Results:**

Participants completed mean 83.9% of EMA surveys (SD = 16.1%). EMA ratings of loneliness were moderately correlated with UCLA loneliness scale scores. Network models showed that loneliness was contemporaneously associated with negative affect (worried, anxious, restless, irritable). Negative (but not happy or positive) mood tended to be followed by loneliness and then by exercise or outdoor physical activity. Negative affect had significant and high inertia (stability).

**Conclusions:**

The data suggest that EMA is feasible and acceptable to older adults. EMA-assessed loneliness was moderately associated with scale-assessed loneliness. Network models in these independent living older adults indicated strong links between negative affect and loneliness, but feelings of loneliness were followed by outdoor activity, suggesting adaptive behavior among relatively healthy adults.

## Introduction

Social Isolation and Loneliness (SI/L) have assumed pandemic proportions over recent decades, in part driven by globalization and ultra-rapid rise in technology (1, 2). The situation has been exacerbated by the ongoing containment measures for the COVID-19 pandemic and mandated lockdowns. The impact could be greater in older adults due to their physical vulnerability (2, 3). However, studies have shown higher levels of resilience and wisdom in older than in younger adults during the pandemic (4). A common inference during COVID-19 pandemic and the ensuing strict isolation measures is that older adults in independent living conditions were likely to have encountered loneliness (5–8); During the pandemic period, it is unclear whether and how day-to-day or micro-level experiences of loneliness related to affect or behavior.

Chronic loneliness is a consistent set of beliefs regarding the lack of connections with others and yet state loneliness refers immediate experience of social disconnection. Under Cacioppo model, state loneliness is not necessarily negative but may motivate behavior such as outreach or seeking social interaction (9, 10). Loneliness and social isolation are weakly correlated (11–13). An individual's relationships such as friends and family may influence activity (14) and social isolation was associated with behavioral inactivity in general (15). The relationship between loneliness and social behavior is somewhat unclear in older adults. Loneliness was not found to be related to social activity among older adults in one study (16). Moreover, the COVID-19 pandemic placed additional restrictions on mobility which further may have altered social behavior. While much is known about chronic loneliness and long-term health effects, the impact of state loneliness on day-to-day behavior is less researched, particularly its dynamics among older adults during the pandemic.

Ecological momentary assessment (EMA) allows for relatively unobtrusive monitoring of affect as well as physical and social context variables, which when monitored repeatedly over time, can uncover dynamic relationships between variables (17). While traditional approaches are limited to discovering associations or correlations, EMA allows one not only to establish the said associations, but also time lags and leads which enable hypotheses for possible causality (18). A recent meta-analysis suggested 81.9% mobile EMA compliance in adults (19). However, to date, use of EMA in the “older-old” adults (persons over age 80) or to study loneliness is somewhat limited, with none focused on loneliness during the pandemic using network models. A broad search in PubMed on EMA in geriatric populations with manual screening of mean age around 70 produced a few results, focused on perception and usability (20–22), and diverse applications included adverse event monitoring (23), Multiple Sclerosis (24) and pain (25). Some EMA studies have included adults with mean ages ranging from 69 to 73, suggesting feasibility (26–28). An EMA study on loneliness in the older population (mean age 73.7) suggested men reported greater intensity of loneliness, and being outdoors lessened the feeling—the effects were weaker among women and non-Whites (29). Another EMA study, not limited to older adults, during COVID-19 lockdown found that a composite “negative-mood” score (comprising fatigue, anxiety, stress, depression and unhappiness) tended to accumulate over time, and the score was positively and significantly associated with COVID19-related worry, the perception of restrictions, and loneliness (30).

EMA studies evaluating lagged associations (e.g., mood associations with subsequent behavior or vice versa) typically evaluated fixed time lags and univariate relationships. However, between-people networks, constructed by combining data from several individuals, allow us to discover multiple contemporaneous and lagged associations representative of the group (17). To our knowledge, this is the first study to apply network models to EMA data to explore the loneliness experience of older adults (mean age 80+) residing in senior housing communities during the COVID-19 pandemic. Due to the older mean age of this sample (80 years) than in prior studies, we evaluated both the feasibility of EMA with respect to adherence and also convergence of EMA questions on loneliness with standard scale-based measures of loneliness. We then applied network models to evaluate sequential relationships and moment-to-moment interactions among emotions, and loneliness, and behavior.

We hypothesized that: (a) Older adults would evidence acceptable (e.g., >75% adherence to EMA procedures, (b) Loneliness as measured by EMA would be significantly associated with an in-lab scale-based measure of loneliness (UCLA Loneliness Scale), and (c) Network models applied to EMA data would reveal significant contemporaneous and lagged connections between momentary loneliness, affect, and social behavior.

## Methods

### Participants

Participants were recruited from an ongoing longitudinal study of older adults aged 65 years and above living independently in a Continued Care Senior Housing Community (CCSHC) (31). Participants were contacted by study staff to assess level of interest. Eligibility requirements included current enrollment in the parent study and access to a smartphone capable of receiving daily text messages and surveys. Parent study exclusion criteria included people with dementia, major mental illness or other conditions that could interfere with study participation and those who are unable to read and write in English. The sample (*n* = 22) included 19 women and three men (Table 1). The EMA surveys were collected between 5/25/2020 and 8/16/2020.

**Table 1:** Socio-demographic and clinical factors (*N* = 22).

	**Mean or %**	**SD**	**Min**	**Max**
**Socio-demographic**				
Age (years)	80.24	7.13	68.2	93.4
Education (years)*	15.59	2.63	12.0	20.0
Race (% Caucasian)*	91%			
Marital Status (% married/co-habitating)	32%			
**Loneliness and social support measures**				
UCLA-3 1st Administration	35.86	7.92	24.0	49.0
UCLA-3 2nd Administration	29.87	5.74	23.0	44.0
UCLA-3 3rd Administration	33.33	9.55	24.0	56.0
UCLA Averaged over all available	34.77	7.86	24.5	49.3
Emotional Support* (ESS-E)	2.74	0.46	1.5	3.0
Instrumental Support* (ESS-I)	1.67	0.83	0.5	3.0
Negative social interactions* (ESS-NI)	0.33	0.43	0.0	1.5
**Clinical measures**				
Depression* (PHQ-9)	2.14	2.41	0.0	8.0
Anxiety* (BSIAS)	1.86	3.48	0.0	12.0
**EMA measures**				
Worried	1.344	0.61	1.0	5.0
Happy	4.040	0.94	1.0	5.0
Anxious	1.616	0.83	1.0	5.0
Restless	1.328	0.61	1.0	5.0
Irritable	1.200	0.51	1.0	5.0
Lonely	1.248	0.54	1.0	5.0
Exercise	1.995	0.92	1.0	5.0
Outdoor	1.733	1.12	1.0	5.0
Social interaction	2.208	1.21	1.0	5.0

*BSIAS, Brief Symptom Inventory Anxiety Scale; ESS-E, Emotional Support Scale—Emotional Support score; ESS-I, Emotional Support Scale—Instrumental Support; ESS-NI, Emotional Support Scale—Negative Interaction Score (32); PHQ-9, Patient Health Questionnaire 9-item (33); UCLA-3, UCLA Loneliness Scale (Version 3)*.

* *Baseline data*.

The study protocol was approved by the UC San Diego Human Research Protections Program (HRPP) and all the participants provided a written informed consent prior to study participation.

### Measures

Assessments included sociodemographic as well as clinical measures of depression (Patient Health Questionnaire, 9-item, or, PHQ-9) (33), anxiety (Brief Symptom Inventory—Anxiety subscale, or, BSI) (34), and UCLA Loneliness scale (Version 3) or UCLA-3 (35) which is a 20-item scale. The tests were administered between 5/25/2020 and 8/16/2020. The scores on UCLA-3 loneliness scale can be interpreted as low (range: 20–34), moderate (range: 35–49), moderately high (range: 50–64), and high (range: 65–80) (36, 37). For descriptive purposes, we also administered the PHQ-9 scale for depression, wherein score ranges from mild (5–9), moderate (10–14), moderately severe (15–19) and severe depression (≥20), respectively, along with the BSI anxiety subscale (34, 38) comprises six items, it is a self-report measure of anxiety that ranges from 0 to 24 with higher scores indicating a greater level of anxiety.

### EMA Procedure

Participants were sent text notifications to their personal smartphones to complete the smartphone-based surveys three times daily for 7 days through the online-based survey platform, Alchemer. Each text notification contained a unique participant link to the study surveys. The daily survey notifications were sent at varying times each day, with a minimum 4-h increment between surveys. Participants received the surveys once in the morning, once in the afternoon, and once at night. Two participants opted out of the morning surveys and requested to receive afternoon and evening surveys only. Upon receiving the link, participants completed EMA questions assessing context, mood, and behaviors. Once the link was delivered, the morning and afternoon surveys stayed active for at least 3 h, until 1 h prior to the next scheduled survey being sent, at which point the survey was closed and no longer accessible. The evening surveys closed at 11:00 p.m. each night. Study surveys were linked to participant's smartphone number and were therefore opened only by the participant's device. Deidentification of participant's data was performed and the data was not stored locally on the devices. Survey data were sent to encrypted, HIPAA-compliant cloud storage in Amazon Web Services (AWS), and responses were recorded even if participants did not complete the entire survey. Real-time access to participant's data and daily progress was available through the AWS system. When three surveys in a row were missed by the participants, they were contacted by the research staff to address any technical difficulties or adherence issues.

Each survey was comprised of the 15 EMA prompts related to the previous 2 h; out of these, the responses to following nine prompts were used in the study:

(1) How worried were you generally? (2) how happy vs. sad were you? (3) how relaxed vs. anxious were you? (4) how fidgety or restless were you? (5) how irritable or easily angered have you been? (6) how lonely were you? (7) how many minutes did you exercise or move regularly? (8) how many minutes did you spend time outdoors? and (9) how many people did you spend time with? All responses were scored on 1–5 scale, interpreted from the lowest to the highest intensity based on the prompt context.

### Statistical Analysis

Pearson's correlation was used to assess correlations between EMA variables and UCLA-3 measures of loneliness.

### Network Analysis

Time-series for the EMA response variables were constructed by splicing together the data for each participant (in the same order across the variables). Tigramite, the python implementation of PCMCI (39) algorithm was used to construct the temporal networks with contemporaneous and lagged edges. Temporal lags up to six sampling intervals (2 days) were analyzed. The implementation is designed to handle some missing data when appropriately tagged. It generates error when an unacceptable amount of data is missing, however, we did not encounter that situation.

Unlike studies based upon effect sizes that draw direct benefit from large sample sizes, small sample correlation-based studies are susceptible to type-1 error, of identifying correlations when none exists in larger population. Since our sample was small (*n* = 22), our network models use PCMCI that incorporates Benjamini–Hochberg Method (40) (also called BH procedure) to limit false discovery rate.

## Results

Demographic and clinical details are presented in Table 1. The mean age of participants was 80.24(SD = 7.13) years, 32% were married or cohabitating. Average loneliness score on UCLA-3 scale was 34.8 (SD = 7.86). Scores for depressive and anxious symptoms indicated minimal severity, well within the normal range. Participants had over 15 years of education on an average (Table 1) and resided in a single continuing care community that had spaces for both socializing and exercising. Of the 22 participants, one reported ethnicity as Asian and one as African American, the rest reported Caucasian.

Average adherence to the EMA surveys was 83.9% (SD = 16.1%) or an average of 17.0 (SD = 3.6) responses out of a total of 21 survey opportunities. Two participants opted out of the morning surveys and requested to only be sent surveys in the afternoon and evening, therefore receiving 14 survey opportunities each. Evening surveys had the highest adherence at 86.4% (SD = 20%), afternoon surveys had the second highest adherence at 84.4% (SD = 19.2%), and morning survey had the lowest adherence at 80.0% (SD = 18.2%). In addition to the high rate of surveys completed (84% of administered) all participants who were approached to participate in EMA surveys enrolled and completed the 7-day protocol. Notably, adherence was worse on the first few days and then improved (Spearman's *r* = 0.33, *p* < 0.001), thus, EMA surveys were not associated with fading or fatigue effects but rather non-adherence problems at the outset that resolved. There were however, two participants who consistently declined to respond to the morning survey but were allowed to continue in the study.

EMA loneliness was associated with UCLA-3 Loneliness Scale (*r* = 0.375). Supplementary Table 1 shows the correlations for the EMA affective variables, with EMA loneliness correlated significantly with positive affect and fidgety/restlessness, but not other affective states.

**Figure 1:**
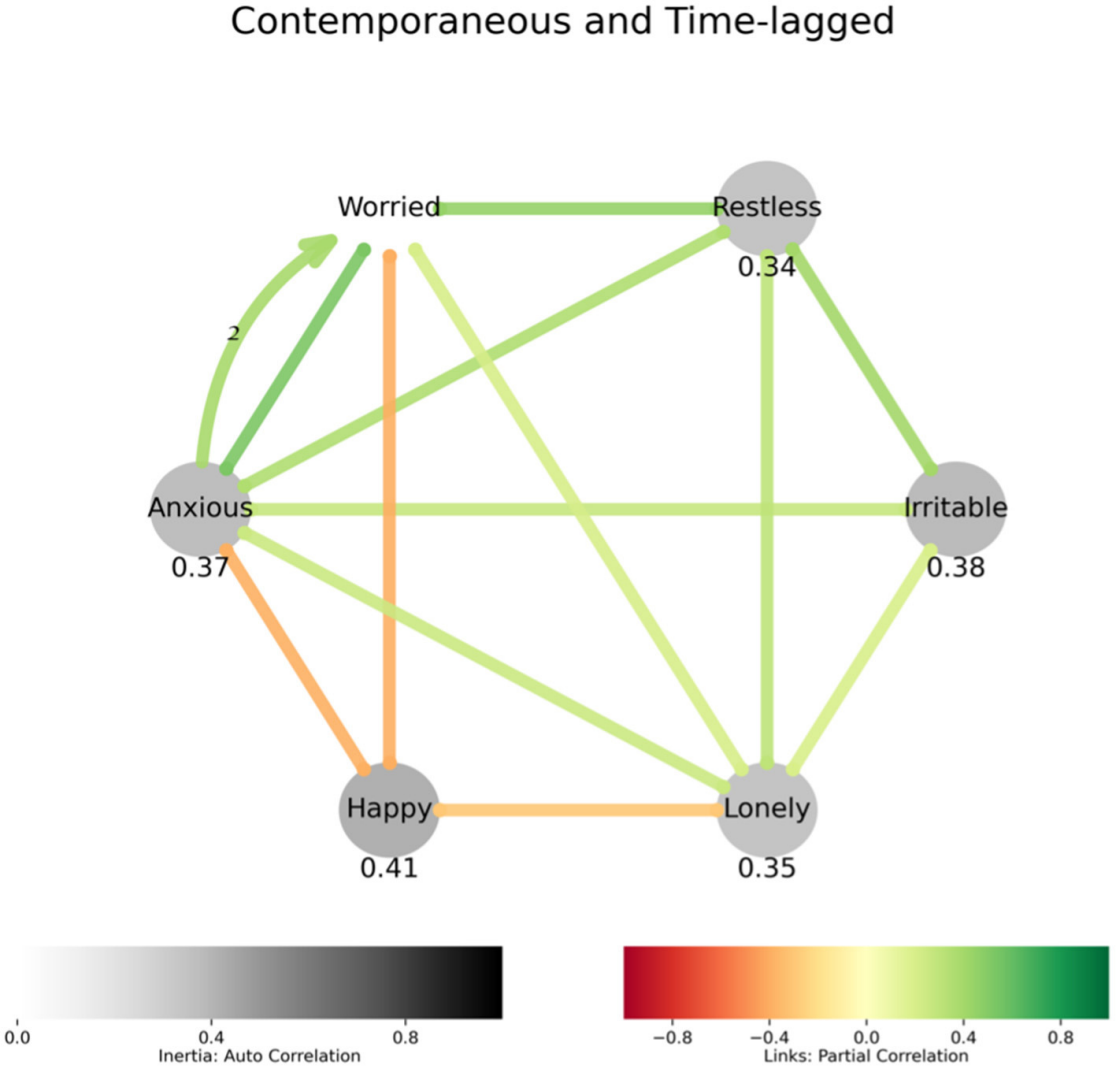
Affect model describing how loneliness relates to affect: Undirected straight edges between variables indicate contemporaneous associations, and the directed labeled arcs represent lagged associations, with the label being the lag in multiples of sampling interval. The colors of the edges and the arcs represent negative (red) or positive (green) association. The variables with gray nodes indicate significant autocorrelation, or inertia, which can be interpreted as the values of these variables showing high resistance to change. If a variable measures polar quantity (happy-sad or relaxed-anxious), the variable is represented in the graph by the label to which higher values are assigned, and “how happy vs. sad were you” is simply “Happy.” Positive associations are in shades of green, and negative in shades of red. Lagged links are curved, have arrowheads and display lag in multiples of 8 h. Negative emotional states are associated with loneliness. Inverse relationship between happy and loneliness is also expected. Anxiety and worry display a positive feedback loop.

**Figure 2:**
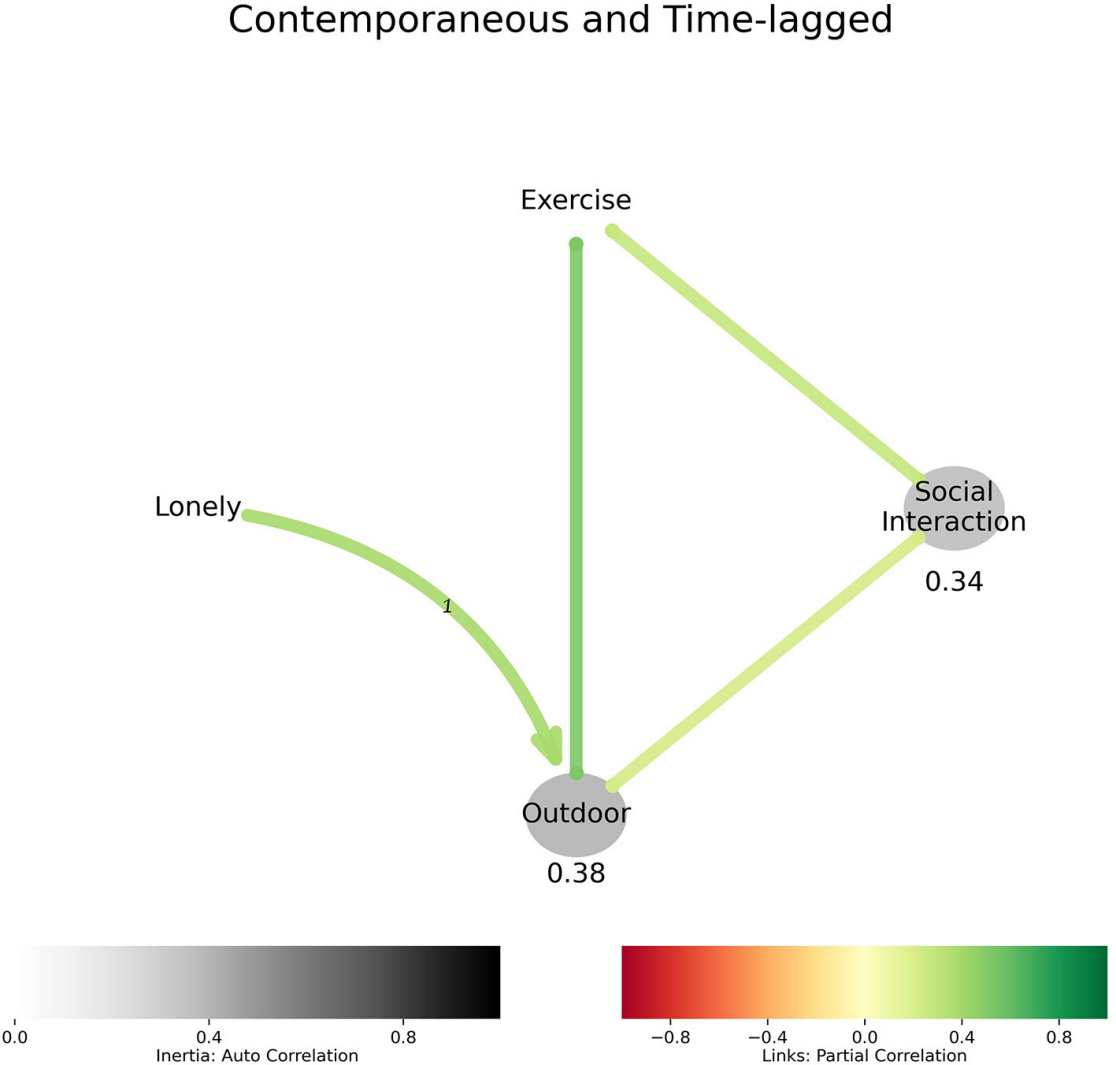
Behavior model describing how loneliness relates to behavior: Loneliness precedes being outdoor, which is associated with exercise and social interaction.

**Figure 3:**
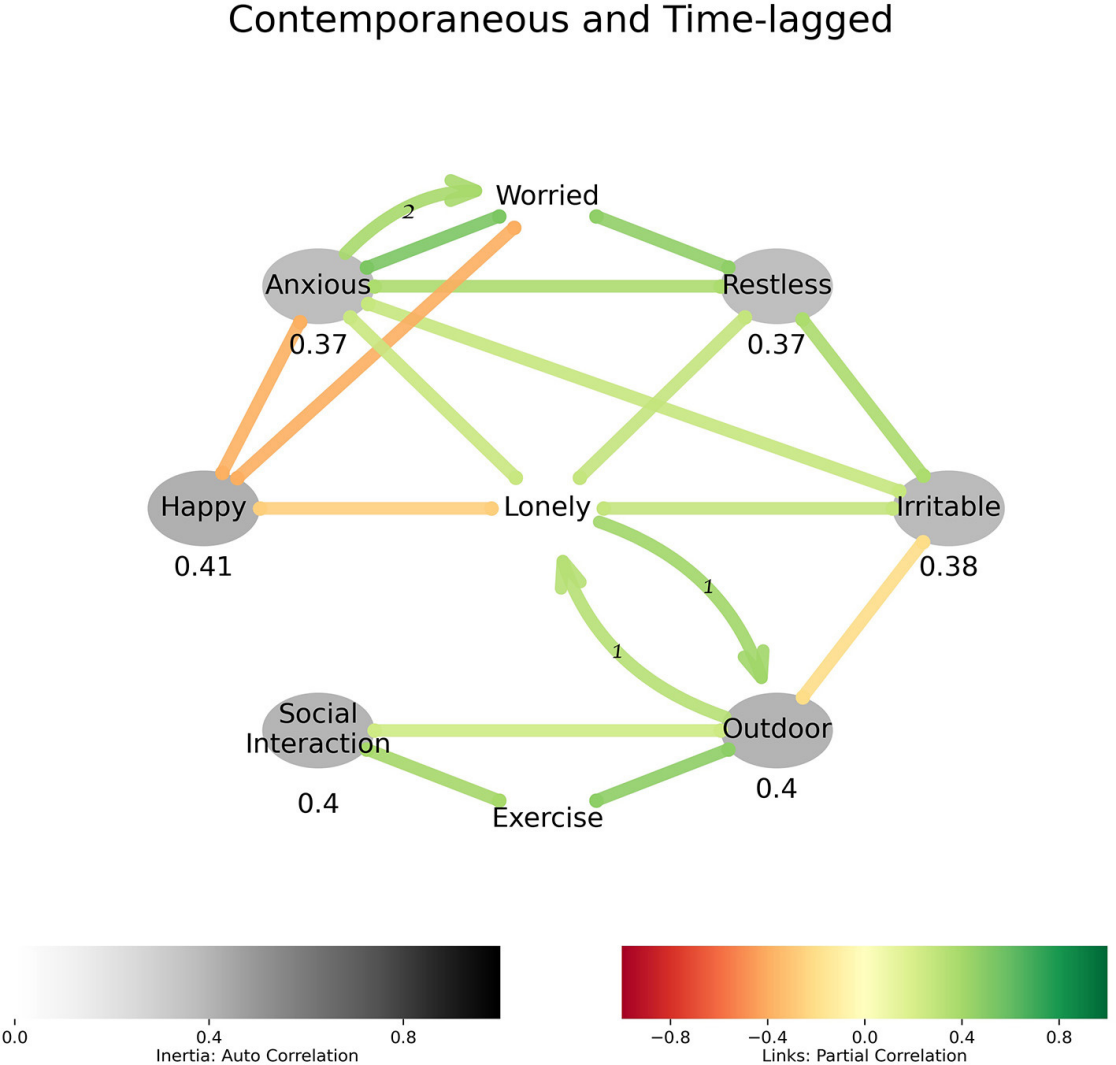
Affect, loneliness, and behavior: Loneliness is predictive of being outdoors. Loneliness is not contemporaneous to being outdoors, it precedes it and returns soon after.

The networks (Figures 1–3) show subsets of variables analyzed, and their lagged and contemporaneous associations. Network analysis of affective experience identified that loneliness was contemporaneously associated with feelings of restlessness, worry, irritability and anxiety and a lack of happiness (Figure 1). A positive feedback loop between anxiety and worry suggests these experiences may converge to increase each other.

Figure 2 evaluated loneliness and resultant behaviors. Loneliness preceded being outdoors in the short-term and being outdoors was contemporaneous with exercise and social interaction in these older adults.

Figure 3 integrates affect and behavior models and shows that loneliness was strongly associated with negative feelings and a general lack of happiness. Being outdoors was associated with lower irritability. Figure 3 also shows a relationship that seemed to exist between being lonely and being outdoors. Since the two did not exist contemporaneously, loneliness can be interpreted as being experienced when indoors. This was followed by an outdoor-seeking adaptive behavior that showed up in the next sampling (a lag of 1τ or, 8 h) when the participant was outdoors. The feeling of loneliness seemed to return soon after returning from outdoors (again, a lag of 1τ or, 8 h), and being outdoors was associated with exercise and social interaction.

## Discussion

We used EMA and dynamic network models to explore loneliness and its behavioral and affective concomitants in a sample of older adults. The primary findings from this study are three-fold; (1) EMA of loneliness and its concomitants was a feasible technique in older adults (mean age 80.2 years) with a sample adherence rate of 83.9%. (2) EMA of momentary loneliness was moderately associated with scale-assessed loneliness (UCLA-3 Loneliness Scale). (3) Network models displayed a variety of links between loneliness, affect and behavior. While loneliness was associated with negative emotions, our results suggest that loneliness was associated with short-term adaptive behavior, in particular spending time outdoors. This temporal finding is supported by another EMA study that being outdoors lessened the feeling of loneliness in the short term (29). These network models point to the need for future research to understand the behavioral sequelae of loneliness, delineating adaptive and maladaptive responses (and the influence of policies on those responses) to acute loneliness as they might contribute to or mitigate chronic loneliness.

The finding that loneliness is associated with negative emotions and diminished happiness is not surprising and is consistent with other studies (41–43), and during the lockdown in particular (30). A potentially novel finding through network models applied to EMA data is that at least some older people may have coped with momentary experiences of loneliness by actively seeking outdoor activity. There was a strong association between being outdoor and exercise, and exercise and social interaction, but a weaker association between outdoor and social interaction in Figure 3. These findings are consistent with the literature indicating that a direct link between loneliness and social interaction behavior is weaker than might be expected (11–13). Since loneliness was not contemporaneous with being outdoors (and its correlates of activity and social interaction), it can be inferred to be associated with lower activity levels in the moment, and subsequent outdoor time. In that sense, acute loneliness, in this relatively healthy sample with a low level of distress, may have led to adaptive social behaviors. It has previously been suggested that loneliness serves a variety of adaptive functions (44). Previous literature also shows that coping mechanisms also differ by severity of depression among older adults, as self-distraction has been shown to be common among people with depression depressed group, while active coping was common among people without depression (45). Furthermore, our results are consistent with emotion/loneliness preceding activity, as in a different study, activity in-and-of itself had little effect on positive or negative affect (46). A study identified going outdoors as a coping strategy for social isolation during the pandemic among adults and included it in the survey (47), however no significant difference was observed in social isolation of those who did and did not seek outdoors. In an online study that included PHQ-9 questions and coping strategies, staying outdoors and looking outside were among the best predictors of lower levels of depressive symptoms associated with COVID-19 related isolation (48). Thus, how acute loneliness intersects with chronic loneliness is an important area for future research; EMA may be useful for contrasting loneliness at different time scales from day-to-day variations to more chronic experiences as well as for identifying which individuals would most likely benefit from specific types of interventions (e.g., those best suited for acute or chronic loneliness).

It was notable that the adaptive response to loneliness in this sample was to go outdoors. Variation in the extent of lockdowns or shelter-in-place guidelines observed during the pandemic may have influenced how people accessed outdoor activity and putatively coped with loneliness. Since this was a single-site study, it is not possible to evaluate variation by outdoor access. Nonetheless, technologically based alternative solutions to provide adaptive opportunities might be considered to help older adults cope with loneliness under circumstances where access to outdoor activity may be restricted.

This study has some limitations, and it should be considered as a preliminary work to test feasibility and explore relationships among study variables for future replication. The sample size was small. The participants were drawn from a single site disallowing analysis of variation by level of restriction. There are also technical aspects of EMA study design that have a strong bearing on the findings, such as sampling interval and duration. Three samplings per day, as in our case, would imply that phenomena lasting less than the sampling interval (24/3 = 8 h) may not be captured in sufficient detail in our network models. More frequent sampling may reveal greater detail; however, it may also easily become intrusive and burdensome to older participants. It should be noted that objective measures of loneliness using UCLA-3 were available at three distinct checkpoints, whereas the subjective measures were a part of EMA sampling—this time gap may have attenuated the correlation between EMA and scale-based loneliness. In understanding the influence of loneliness on behavior, it is important to account for concurrent depressive symptoms. This sample had very low levels of depression on average, and so these results may not generalize to samples with greater variation in depressive symptoms. Lastly, the study was performed during the early period of COVID-19 pandemic (between 5/25/2020 and 8/16/2020) and before the FDA approval of first vaccine, the social-distancing rules may have altered the living conditions and limited the activities of the cohorts.

In conclusion, EMA-based network modeling appears to be a useful tool for assessing momentary loneliness in older adults. Given issues with early adherence that later resolved, follow-up with participants at the outset of EMA survey protocols may support adherence. Our study points to potentially important nuances to understanding the connection between acute loneliness and behavior, and how policy and environmental influences may impact response to short-term loneliness. Future study should examine how momentary loneliness, day-to-day behavior and affective experience converge to contribute to chronic loneliness, such as in a measurement burst design (49). This technique uses *bursts* of frequently repeated assessments in a short period of time, spanning a few days or weeks. Such burst measurements are repeated longitudinally over a longer interval (after a few months or a year), capturing not only individual differences, but also the short-term variability in measured variables and long-term trends, *vis a vis* chronic loneliness and its impact on health over the course.

## Data Availability Statement

De-identified data supporting the conclusions of this article will be made available by the authors to qualified investigators. Further queries can be directed to the corresponding author/s.

## Ethics Statement

The studies involving human participants were reviewed and approved by UCSD Human Research Protections Program. The patients/participants provided their written informed consent to participate in this study.

## Author Contributions

CAD, VDB, and EEL contributed to conception and design of the study. RD organized the database and supported the data collection activity. VDB performed the network analysis and CAD oversaw the study. VDB wrote the first draft of the manuscript. CAD, VDB, H-CK, EMP, EEL, RD, and DVJ edited and contributed to the manuscript. All authors contributed to manuscript revision, read, and approved the submitted version.

## Funding

This work was supported by IBM Research AI through the AI Horizons Network. This study was supported, in part, by the National Institute of Mental Health [NIMH T32 Geriatric Mental Health Program MH019934 (DVJ), K23 grant-MH119375-01 to EEL, by the Stein Institute for Research on Aging (DVJ) and Veterans Affairs Healthcare System.

## Author Disclaimer

The content of this paper is solely the responsibility of the authors and does not necessarily represent the official views of the NIH.

## Conflict of Interest

H-CK is an employee of IBM. The remaining authors declare that the research was conducted in the absence of any commercial or financial relationships that could be construed as a potential conflict of interest.

## Publisher's Note

All claims expressed in this article are solely those of the authors and do not necessarily represent those of their affiliated organizations, or those of the publisher, the editors and the reviewers. Any product that may be evaluated in this article, or claim that may be made by its manufacturer, is not guaranteed or endorsed by the publisher.
