# Supplementary material for: Dynamics of Loneliness Among Older Adults During the COVID-19 Pandemic: Pilot Study of Ecological Momentary Assessment With Network Analysis
Source: Front Digit Health. 2022 Feb 7;4:814179. doi: 10.3389/fdgth.2022.814179 (PMC8859335; doi:10.3389/fdgth.2022.814179)
Supplement: Supplementary file 1 [file Data_Sheet_1.PDF]

Supplementary Table 1: EMA Pilot Data Pearson's Correlations

	Worried		Happiness		Anxiety		Restlessness		Fatigue		Lack of Concentration		Irritability		Loneliness	
	r	***p***	***r***	***p***	***r***	***p***	***r***	***p***	***r***	***p***	***r***	***p***	***r***	***p***	***r***	***p***
How worried were you?			-.432*	0.045	.473*	0.026	0.382	0.080	0.197	0.379	0.180	0.422	.458*	0.032	0.054	0.811
How happy vs. sad were you?	-.432*	0.045			-.696**	0.000	-.614**	0.002	-.525*	0.012	-.663**	0.001	-.633**	0.002	-.559**	0.007
How relaxed vs. anxious were you?	.473*	0.026	-.696**	0.000			.849**	0.000	.435*	0.043	.629**	0.002	.760**	0.000	0.360	0.100
How fidgety or restless were you?	0.382	0.080	-.614**	0.002	.849**	0.000			0.149	0.508	0.397	0.068	.609**	0.003	.468*	0.028
How fatigued/tired were you?	0.197	0.379	-.525*	0.012	.435*	0.043	0.149	0.508			.677**	0.001	.516*	0.014	0.372	0.088
How were you able to concentrate/focus?	0.180	0.422	-.663**	0.001	.629**	0.002	0.397	0.068	.677**	0.001			.654**	0.001	0.363	0.097
How irritable were you?	.458*	0.032	-.633**	0.002	.760**	0.000	.609**	0.003	.516*	0.014	.654**	0.001			0.421	0.051
How lonely were you?	0.054	0.811	-.559**	0.007	0.360	0.100	.468*	0.028	0.372	0.088	0.363	0.097	0.421	0.051		

* Correlation is significant at the 0.05 level (2-tailed).
** Correlation is significant at the 0.01 level (2-tailed).

Worried: Positively correlated with anxiety and irritability; negatively correlated with happiness
Happiness: Negatively correlated with worry, anxiety, restlessness, fatigue, concentration, irritability, and loneliness
Anxiety: Positively correlated with worry, restlessness, fatigue, lack of concentration, and irritability; negatively correlated with happiness
Restlessness: Positively correlated with anxiety, irritability and loneliness; negatively correlated with happiness
Fatigue: Positively correlated with anxiety, lack of concentration, and irritability; negatively correlated with happiness
Lack of concentration: Positively correlated with anxiety, fatigue, and irritability; negatively correlated with happiness
Irritability: Positively with correlated worry, anxiety, restlessness, fatigue, and lack of concentration; negatively correlated with happiness
Loneliness: Positively correlated with restlessness; negatively correlated with happiness
